# Draft Genome Sequence of the Iridescent Marine Bacterium Tenacibaculum discolor Strain IMLK18

**DOI:** 10.1128/MRA.01683-18

**Published:** 2019-01-31

**Authors:** H. Lynn Kee, Irina V. Mikheyeva, Rebecca L. Mickol, Scott C. Dawson, Dianne K. Newman, Jared R. Leadbetter

**Affiliations:** a Department of Biology, Stetson University, Deland, Florida, USA; b Department of Microbiology and Immunology, Geisel School of Medicine at Dartmouth, Hanover, New Hampshire, USA; c American Society for Engineering Education, Washington, DC, USA; d Department of Microbiology and Molecular Genetics, College of Biological Science, University of California, Davis, Davis, California, USA; e Division of Geological and Planetary Sciences, The California Institute of Technology, Pasadena, California, USA; f Division of Biology & Biological Engineering, The California Institute of Technology, Pasadena, California, USA; g Divisions of Engineering & Applied Science, The California Institute of Technology, Pasadena, California, USA; University of Maryland School of Medicine

## Abstract

We report here the draft genome sequence of a strain of Tenacibaculum discolor (*Bacteroidetes*) that was isolated from the river-ocean interface at Trunk River in Falmouth, Massachusetts. The isolation and genomic sequencing were performed during the 2016 and 2018 Microbial Diversity summer programs at the Marine Biological Laboratory in Woods Hole, Massachusetts.

## ANNOUNCEMENT

Tenacibaculum discolor strain IMLK18 was isolated from a seawater sample collected from the river-ocean interface between the Trunk River outlet and Vineyard Sound (Atlantic Ocean) in Falmouth, Massachusetts, in July 2016. A dilution of the seawater sample was cultured on sea water complete (SWC) agar at 25°C for 1 week. One colony exhibited iridescence in hues of green and orange; this was streaked for purity via three successive clonal picks. Surface translocation by gliding motility was observed. Strain IMLK18 is composed of rods of 1 μm in length. The 16S rRNA gene sequence of strain IMLK18 was analyzed after colony PCR amplification and Sanger DNA sequencing; it was 94% similar to that of T. discolor strain LL04 11.1.1. The LL04 11.1.1 strain of T. discolor was obtained from the kidney of a specimen of sole (Solea senegalensis, from Galicia, Spain) that displayed characteristics of marine fish disease flexibacteriosis, e.g., rotten mouth and fin and skin lesions (1).

The original isolate was stored at −80°C in SWC and 15% glycerol, and from this, a culture was grown on SWC agar and used to grow an overnight culture at 30°C in SWC broth. Genomic DNA was extracted from the pelleted cells from the liquid culture using the Maxwell RSC PureFood genetically modified organism (GMO) and authentication kit (Promega) and quantified using the double-stranded DNA (dsDNA) QuantiFluor ONE kit (Promega) using the Quantas system (Promega). Libraries were generated using the Nextera DNA flex library kit. Genome sequencing was conducted using the Illumina HiSeq 2500 platform (2 × 250-nucleotide [nt] paired-end reads). FastQC was utilized to assess the quality of the sequence reads. Genome assembly of 2,948,886 paired reads was performed using SPAdes 3.12.0 (2). The genome consists of 109 contigs yielding a total length of 3,388,982 bp, an *N*_50_ contig size of 431,359 bp, and coverage of 441×. The average G+C content was 31.60%. The average nucleotide identity (ANI) (3) value of 98.23% to the genome of T. discolor strain DSM 18842 is above the criterion (95%) for assignment to the same species, which indicates that strain IMLK18 is a new strain of T. discolor. Genome annotation was completed using Prodigal 2.6.1 (4) and resulted in 3,285 coding sequences (5). Gene annotation reveals the presence of genes involved in gliding motility and the type IX secretion system, including *gldA* and *sprF* that were previously shown to result in iridescent defects when disrupted in the bacterium Flavobacterium sp. strain IR1 (6, 7). Further research will provide insights into mechanisms of biological iridescence, as well fish pathogenicity by marine Bacteroidetes species. The genome sequence of T. discolor strain IMLK18 will improve our understanding and facilitate future comparative studies of the genus Tenacibaculum.

### Data availability.

The GenBank accession number for this genome sequence is RCVH00000000, and the SRA accession number for the Illumina sequencing run is SRS4170023.
